# Trend in Obesity Prevalence in European Adult Cohort Populations during Follow-up since 1996 and Their Predictions to 2015

**DOI:** 10.1371/journal.pone.0027455

**Published:** 2011-11-10

**Authors:** Anne von Ruesten, Annika Steffen, Anna Floegel, Daphne L. van der A, Giovanna Masala, Anne Tjønneland, Jytte Halkjaer, Domenico Palli, Nicholas J. Wareham, Ruth J. F. Loos, Thorkild I. A. Sørensen, Heiner Boeing

**Affiliations:** 1 Department of Epidemiology, German Institute of Human Nutrition Potsdam- Rehbruecke, Nuthetal, Germany; 2 Centre for Nutrition and Health (CVG), National Institute for Public Health and the Environment (RIVM), Bilthoven, The Netherlands; 3 Molecular and Nutritional Epidemiology Unit, Cancer Research and Prevention Institute – ISPO, Florence, Italy; 4 MRC Epidemiology Unit, Institute of Metabolic Science, Cambridge, United Kingdom; 5 Danish Cancer Society, Institute of Cancer Epidemiology, Copenhagen, Denmark; 6 Institute of Preventive Medicine, Copenhagen University Hospital, Copenhagen, Denmark; Bremen Institute of Preventive Research and Social Medicine, Germany

## Abstract

**Objective:**

To investigate trends in obesity prevalence in recent years and to predict the obesity prevalence in 2015 in European populations.

**Methods:**

Data of 97 942 participants from seven cohorts involved in the European Prospective Investigation into Cancer and Nutrition (EPIC) study participating in the Diogenes project (named as “Diogenes cohort” in the following) with weight measurements at baseline and follow-up were used to predict future obesity prevalence with logistic linear and non-linear (leveling off) regression models. In addition, linear and leveling off models were fitted to the EPIC-Potsdam dataset with five weight measures during the observation period to find out which of these two models might provide the more realistic prediction.

**Results:**

During a mean follow-up period of 6 years, the obesity prevalence in the Diogenes cohort increased from 13% to 17%. The linear prediction model predicted an overall obesity prevalence of about 30% in 2015, whereas the leveling off model predicted a prevalence of about 20%. In the EPIC-Potsdam cohort, the shape of obesity trend favors a leveling off model among men (R^2^ = 0.98), and a linear model among women (R^2^ = 0.99).

**Conclusion:**

Our data show an increase in obesity prevalence since the 1990ies, and predictions by 2015 suggests a sizeable further increase in European populations. However, the estimates from the leveling off model were considerably lower.

## Introduction

Obesity (BMI ≥ 30 kg/m^2^) is associated with a high risk of chronic diseases such as diabetes, cardiovascular disease, certain cancers and thus constitutes a major public health problem [1]. In the past, the prevalence of obesity has been increasing rapidly in Europe and all over the world and is estimated to increase further [2], [3], [4]. However, the development of obesity differs across countries [1], [5] and across subpopulations within countries [6].

To achieve the most accurate prediction of future obesity prevalence is a significant public health concern, especially for health services and health economic reasons in the developed countries. Previously, most researchers based their predictions on the assumption that the trend is linear [7], [8], [9]. The assumption of a linear trend of increase of the prevalence in obesity over a longer time period in the future, however, could result in unrealistic scenarios.

Moreover, most studies on obesity trends are based on cross-sectional survey data [2], [10], [11], [12]. Cross-sectional data, however, has the disadvantage that they only display the status at a certain moment, but do not show the dynamics with respect to age, period and birth cohort. Only data from longitudinal studies can address such issues. Despite this, longitudinal data on obesity trends are rarely found in the literature compared to cross-sectional data.

In view of the importance of understanding the dynamics of the obesity epidemic, we used longitudinal data from five European countries participating in the Diet, Obesity and Genes (DiOGenes) [13] project to investigate the current state and observed time trends of obesity prevalence. Furthermore, we aimed to predict the prevalence in 2015 based on the observed trends assuming that the trends observed in recent years will continue. The prediction includes a linear and non-linear projection assuming a leveling off of the obesity trend in the latter model. Both models were evaluated at the EPIC-Potsdam study with five weight measurements over time.

## Materials and Methods

### Study Populations

#### Diogenes cohort

The Diogenes cohort included participants from five countries involved in the European Prospective Investigation into Cancer and Nutrition (EPIC)-study, namely Italy (Florence), the UK (Cambridge), The Netherlands (Amsterdam-Maastricht and Doetinchem), Germany (Potsdam) and Denmark (Aarhus and Copenhagen). The EPIC study has been approved by each local ethics committee of all participating study centers. The International Agency for research on Cancer (IARC) ethics committee (IEC) gave its final vote based on all ethic votes of the local ethic committees. Written informed consent for measurements and inquiries including prospective data collection has been obtained from all participants before joining EPIC. Detailed information on the study population and data collection of the EPIC study has been described elsewhere [14].

Between 1992 and 1998, 146 543 participants were recruited, from which 102 346 (69.8%) took part in the follow-up examination during 1998-2005. We excluded subjects with missing dietary or follow-up data, women who were pregnant at baseline or follow-up, subjects who were in the top and bottom 1% energy intake/energy expenditure ratio, and those with implausible anthropometric measures. Furthermore subjects with missing date of recruitment were excluded; eventually 97 942 persons (41 456 men and 56 486 women) were available for the final analysis. Due to differences in anthropometric measurements at follow-up and length of follow-up time, participants from Doetinchem were separated from those in Amsterdam and Maastricht; thus, a total of seven cohorts were included in the present analysis.

#### EPIC-Potsdam

The EPIC- Potsdam cohort, which also contributed to the Diogenes cohort, consists of 27 548 participants from the general population of Potsdam, Germany, that were aged mainly 35–65 years at recruitment (1994–1998) [15]. Every 2-3 years, updated information on body weight was available by follow-up questionnaires [16], [17]. Response rates for each follow-up round exceeded 90%. Five measurements of weight during the observation period were available for each participant. Subjects with missing information for weight, height, or those who died or dropped out during the follow-up period were excluded from this study. In total, 22 229 participants (8 438 men and 13 791 women) with information for weight at every follow-up assessment were included into this study. Participants were followed until the 4^th^ period of follow-up (2004–2008).

### Anthropometric measurements

Data on body weight was collected at the baseline and follow-up examinations. Height and weight were measured according to centre-specific protocols. In Florence (I), Potsdam (D), Copenhagen and Aarhus (DK) weight was measured on participants in light underwear to the nearest 0.1 kilograms whereas in Amsterdam-Maastricht (NL), Doetinchem (NL) and Cambridge (UK) participants were weighted to the nearest 0.1 kilograms in light clothing after the removal of shoes. Details of the methods of anthropometric measurement used at baseline have been described previously [18]. At follow-up, body weight was directly measured on participants in light clothing in the UK and Doetinchem (NL), whereas in the other centers body weight was assessed based on self-reports. In the Diogenes cohort, which refers to a combined dataset of seven EPIC centers from five countries, one follow-up measurements of weight was available for each participant while in the single dataset of the EPIC-Potsdam cohort four follow-up weight assessments were available.

Referring to body weight, corrections for differences in clothing at baseline and follow-up as well as corrections for self-reporting at follow-up were applied to consider differences in anthropometric assessment among the centers and to increase comparability. To correct for clothing, 1 kilogram was subtracted for weight if participants were weighed in light clothing. Corrections for self-reporting at follow-up were done using calibration methods developed previously in the EPIC study [18].

BMI was calculated based on body weight and height (weight (kg)/height (m)^2^) and, according to current guidelines [1], overweight and obesity were defined as a BMI of 25.0–29.9 kg/m^2^ and ≥ 30 kg/m^2^, respectively.

**Statistical Analysis**

#### Development of obesity prevalence among the Diogenes cohort

The magnitude of increase in obesity prevalence between baseline and follow-up examinations was assessed by comparing the prevalence of obesity (BMI ≥ 30 kg/m^2^) at baseline with that at follow-up. All analyses were conducted separately for men and women. Moreover, stratification due to study centre and age group at baseline was applied to consider the effect of age as well as differences between the single Diogenes sub-cohorts.

#### Prediction of future obesity development among the Diogenes cohort

The projections of obesity in the near future were calculated based on the two kinds of logistic regression models using individual weight change over time.

In the first model, probability of obesity was considered as dependent variable, and follow-up time and age at recruitment as independent variables. The second model was analogous to the first, with the difference that the follow-up time variable was log-transformed to simulate a non-linear (leveling off) trend in obesity prevalence. All logistic regression analyses were stratified by sex and centre, such that a set of sex- and centre-specific model equations to predict the probability of obesity was derived.

By using these model equations, the probability of each individual of becoming obese (above the threshold of BMI ≥ 30 kg/m^2^) at a certain point in time in the future was estimated. The year 2015 was taken for this analysis which is in the near future to avoid an extrapolation too far beyond the observation period which is likely to result in an unrealistic prediction. The individual values of follow-up time (from recruitment until 2015 (July, the 1^st^)) and age at recruitment were inserted into the respective model equation to extrapolate from the baseline probability of being obese into the future. To estimate the projected prevalence of obesity at the population level, the mean of the individual values was taken.

Due to variation in age distribution between the Diogenes centers, the analysis of trends in obesity prevalence was restricted to the 40–65 y age groups - which in general existed in every sub-sample- to increase comparability between centers. Thus, data from 85 159 individuals were available for this analysis. The effect of age was considered in two ways: 1) age at recruitment was included as a covariate in the prediction models, and 2) trends in obesity were stratified according to specific age groups (40–49, 50–59 and 60–65 years) within the population of 40-65 year old subjects.

#### Investigating the shape of obesity development in EPIC-Potsdam

Additionally, the EPIC-Potsdam dataset was used to investigate which of the two prediction models (linear and non-linear; non-linear means that the increase in obesity is showing a leveling off/saturation level) gives the better model fit. The EPIC-Potsdam study comprises five repeated weight assessments per subject whereas only two weight assessments per subject were available in the other centers. For graphical illustration, the linear and log function were fitted to the five data points (based on Least Square Means) in a simple linear regression model and the coefficients of determination (R^2^) were calculated.

Furthermore, the observed prevalence and the estimated values of logistic regression models for every follow-up period were computed.

## Results

### Development of obesity prevalence in Diogenes

The Diogenes cohort had a mean age of 53.9 years (quartile range: 50.5–59.7 years) and a mean BMI of 25.7 kg/m^2^ (quartile range: 23.0–27.8 kg/m^2^) at baseline. The average follow-up time was 6.4 years (quartile range 5.1–8.3 years). At baseline, 13% of 41 456 men and 13% of 56 486 women were obese, a further 50% of men and 33% of women were overweight (BMI: 25–29.9 kg/m^2^). At follow-up, 17% were obese in men and in women, and 54% of men and 37% of women were overweight.

**Figure 1:**
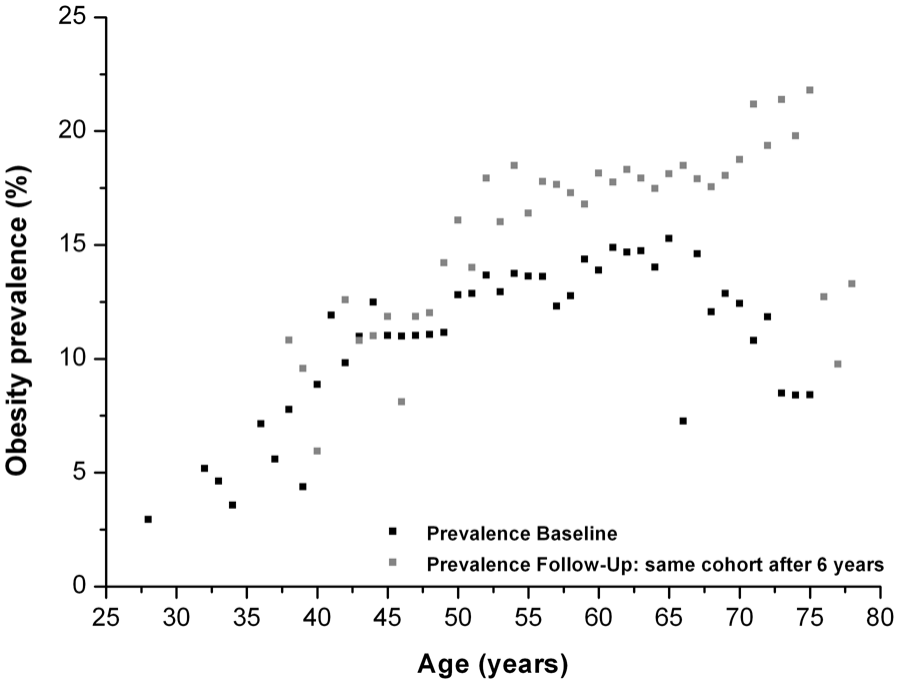
Association of obesity prevalence and age among men of theDiogenes cohort. BMI values of individuals of the total Diogenes cohort used for presenting obesity prevalence depending on the age (only prevalence estimates with at least 100 observations per age were considered for the graph).

**Figure 2:**
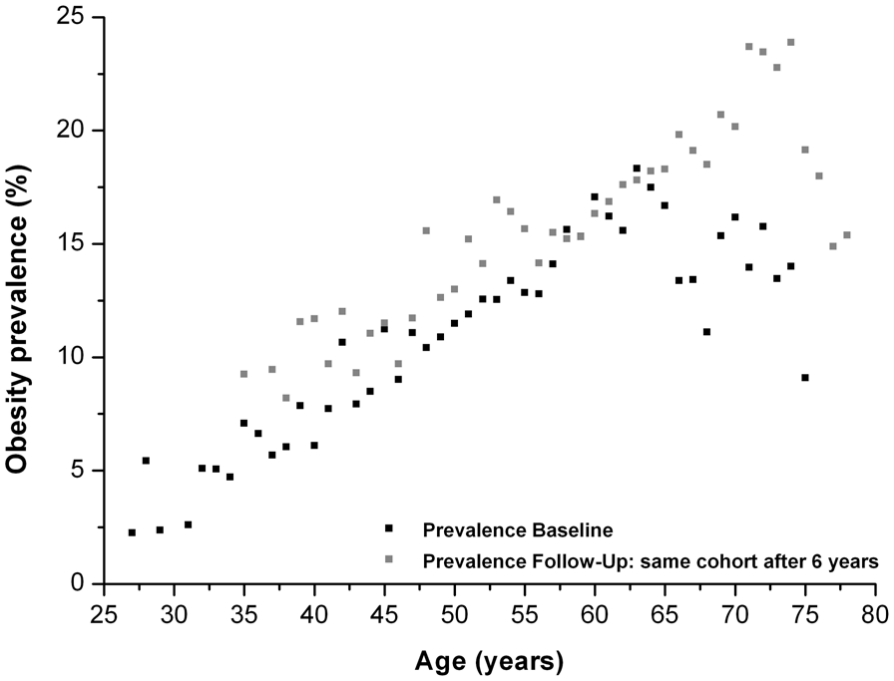
Association of obesity prevalence and age among women of the Diogenes cohort. BMI values of individuals of the total Diogenes cohort used for presenting obesity prevalence depending on the age (only prevalence estimates with at least 100 observations per age were considered for the graph).

In cross-sectional analyses, we observed a strong increase of obesity prevalence with rising age, especially among women between the age of 30 and 65 years (**Figure 1** and **Figure 2**). However, these curves differ between baseline and follow-up, particularly for the 65–75 year age group. Whereas in the initial baseline data lower prevalences could be observed among the 65–75 year age group than in the younger age groups, the high level of obesity prevalence at follow-up persisted even at this old age. The positive relationship between age and obesity prevalence was also observed in most of the individual centers, although the magnitude of increase differed. The increase of obesity prevalence with rising age was especially marked in Amsterdam-Maastricht, Florence and Potsdam in both sexes as well as in Doetinchem and the Danish centers among females (data not shown).

Besides an age effect, we also observed an effect of ‘birth cohort’ on obesity prevalence; i.e. subjects who were in the age group of per example 60 years at recruitment, and shifted to the age group of 65–70 years at follow-up, also showed an increase in obesity prevalence during the follow-up period.

Finally, also when comparing persons of the same age at the baseline and follow-up examination a slight increase in obesity prevalence could be observed, which is more distinctive among males compared to females (**Figure 1** and **Figure 2**).

### Obesity prevalence prediction for 2015 in Diogenes

The simple linear prediction model in 40-65 year old subjects suggested an obesity prevalence of about 30% by year 2015 (range: 20.1–42.7%), whereas the leveling off model predicted an obesity prevalence of about 20% (range: 15.5–27.3%) (**Table 1**). The difference in predicted values from the linear vs. the leveling off model was especially high in centers with a pronounced increase in obesity prevalence during a comparatively short period of follow-up (e.g. Cambridge and Doetinchem). For these centers, extrapolation into the future was associated with a higher degree of uncertainty compared to centers with longer follow-up. When excluding Cambridge and Doetinchem the overall prevalence estimates for 2015 did not change markedly which is due to the relatively small sample size in these cohorts (data not shown).

**Table 1:** Prediction of obesity prevalence (BMI ≥ 30 kg/m^2^) in the Diogenes centers a.

	n	Mean year of baseline measurement	Baseline Obesity [%]	Mean Follow-Up time (in years)	Follow Up Obesity [%]^b^	Prediction 2015: Obesity [%]		Absolute difference between linear and leveling off model in % d
						Linear model c	Leveling off model c	
**Men**								
Potsdam (D)	6 690	1996	16.0	8.6	24.2	36.8	27.3	9.5
Florence (I)	2 048	1995	12.1	9.4	18.3	26.0	20.3	5.7
Cambridge (UK)	4 278	1995	9.2	3.6	12.4	37.8	16.5	21.3
Amsterdam-Maastricht (NL)	2 110	1995	10.8	9.9	17.3	26.4	19.6	6.8
Doetinchem (NL)	1 556	1995	8.7	5.0	13.0	36.4	16.9	19.5
Aarhus (DK)	6 410	1996	13.7	5.3	17.1	29.4	19.7	9.7
Copenhagen (DK)	13 839	1996	13.7	5.4	17.3	30.9	20.1	10.8
Total	36 931	1996	13.1	6.2	17.8	32.2	20.8	11.4
**Women**								
Potsdam (D)	8 690	1996	17.6	8.6	24.4	34.4	26.9	7.5
Florence (I)	6 699	1995	11.4	9.4	17.8	27.3	20.2	7.1
Cambridge (UK)	5 921	1995	12.2	3.6	15.8	40.0	20.2	19.8
Amsterdam-Maastricht (NL)	2 570	1995	10.0	9.9	17.6	28.7	20.3	8.4
Doetinchem (NL)	1 587	1995	12.2	5.0	17.4	42.7	22.0	20.7
Aarhus (DK)	6 780	1996	12.4	5.3	14.2	20.1	15.5	4.6
Copenhagen (DK)	15 981	1996	13.1	5.4	15.0	21.9	16.5	5.4
Total	48 228	1996	13.3	6.5	17.3	27.9	19.6	8.3

a Analysis was restricted to participants who were aged 40-65 y at recruitment.

b One weight assessment was available for every center. Weight was directly measured on participants in light clothing in Cambridge (UK) and Doetinchem (NL) while in the other centers weight was assessed based on self-reports.

c Model uses two points (baseline and follow-up).

d Difference was calculated from subtracting the obesity prevalence for 2015 predicted by the leveling off model from the predicted value for 2015 of the linear model.

In general, obesity prevalences at baseline and at follow-up were higher in women than in men, except of Florence and the Danish centers (**Table 1**).

According to the leveling off scenario, the highest prevalence for 2015 was predicted for men of the Potsdam cohort (27%), whereas the lowest prevalence was estimated for women in the Danish centers (16%). Hence, the estimated absolute increase of obesity prevalence from baseline in the years 1995/1996 until 2015 ranged from 3% (Danish women) and 11% (Potsdam men).

Stratification according to age group at baseline revealed that the increase in obesity prevalence is relatively independent of age, suggesting that the trend is also persistent among the elderly (**Table 2**). Moreover, in most study centers the observed as well as the predicted obesity prevalence was higher in the age group of 60-65 years than in younger age groups.

**Table 2:** Age-stratified prediction of obesity prevalence (BMI ≥ 30 kg/m^2^) for the Diogenes centers a.

	Age at recruitment (y)	n	Baseline %	Follow-Up %	prediction 2015: obesity [%]		Absolute difference between linear and leveling off model in % c
					Linear model b	Leveling off model b	
**Men**							
Potsdam	40-49	2 549	14.7	22.7	35.8	25.9	9.9
	50-59	2 765	14.4	23.7	38.8	27.4	11.4
	60-65	1 376	21.5	27.8	36.1	30.0	6.1
Florence	40-49	916	9.9	17.2	28.0	19.9	8.1
	50-59	868	13.0	18.4	24.3	20.1	4.2
	60-65	264	16.7	21.2	26.0	22.6	3.4
Cambridge	40-49	1 122	7.6	10.6	36.1	14.6	21.5
	50-59	2 016	9.8	12.7	36.4	16.5	19.9
	60-65	1 140	9.8	13.5	42.2	18.3	23.9
Amsterdam-Maastricht	40-49	1 020	9.5	15.9	25.6	18.3	7.3
	50-59	1 090	11.9	18.5	27.2	20.8	6.4
	60-65	---	---	---	---	---	
Doetinchem	40-49	770	7.3	11.9	42.1	16.7	25.4
	50-59	568	10.4	13.7	29.6	16.6	13.0
	60-65	218	9.6	14.7	40.5	19.2	21.3
Aarhus	40-49	----	---	---	---	---	
	50-59	4 971	13.7	17.1	29.3	19.7	9.6
	60-65	1 439	13.6	17.0	29.6	19.6	10.0
Copenhagen	40-49	---	---	---	---	---	
	50-59	10 253	13.7	17.5	31.9	20.3	11.6
	60-65	3 586	13.7	17.0	28.3	19.3	9.0
**Women**							
Potsdam	40-49	3 390	12.5	19.9	32.0	22.8	9.2
	50-59	3 643	18.4	25.0	34.6	27.5	7.1
	60-65	1 657	26.3	32.2	40.2	34.3	5.9
Florence	40-49	1 888	6.3	13.3	26.8	16.4	10.4
	50-59	3 814	13.4	19.4	27.8	21.5	6.3
	60-65	997	13.4	20.3	29.0	22.6	6.4
Cambridge	40-49	1 788	9.7	12.4	29.9	15.6	14.3
	50-59	2 800	12.2	16.2	45.8	21.4	24.4
	60-65	1 333	15.6	19.6	43.3	24.1	19.2
Amsterdam-Maastricht	40-49	1 359	8.0	15.4	27.0	18.2	8.8
	50-59	1 210	12.1	19.9	30.6	22.6	8.0
	60-65	(1)	---	---	---	---	
Doetinchem	40-49	824	8.0	12.4	38.2	16.6	21.6
	50-59	557	17.1	22.6	45.7	27.2	18.5
	60-65	206	15.5	23.3	59.7	30.4	29.3
Aarhus	40-49	---	---	---	---	---	
	50-59	5 423	11.9	14.0	21.3	15.5	5.8
	60-65	1 537	14.1	14.8	16.9	15.3	1.6
Copenhagen	40-49	---	---	---	---	---	
	50-59	11 699	12.3	14.5	22.4	16.1	6.3
	60-65	4 282	15.4	16.6	20.9	17.5	3.4

a Age stratification was used alternatively for adjustment for baseline age in the prediction models (thus only follow-up time was taken as the independent variable).

Age group of 40-49 year-old participants not present in the Danish centers. Age group of 60-65 y was not available in Amsterdam-Maastricht.

b Model uses two points (baseline and follow-up).

c Difference was calculated from subtracting the obesity prevalence for 2015 predicted by the leveling off model from the predicted value for 2015 of the linear model

### Shape of trends in obesity prevalence (EPIC-Potsdam data)

**Figure 3:**
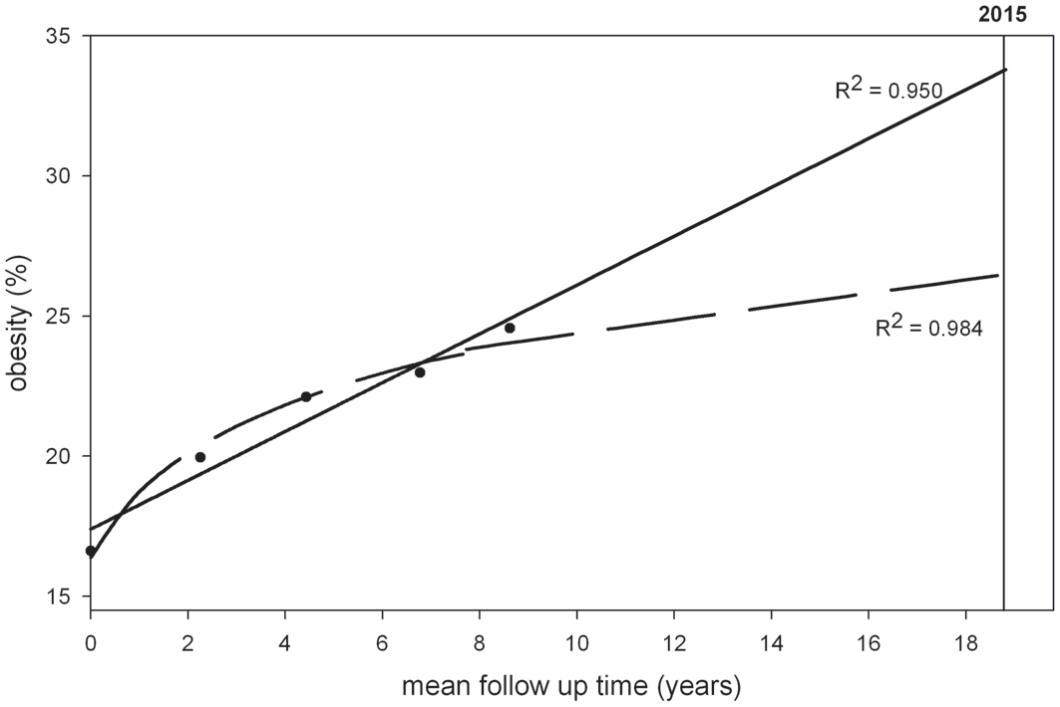
Obesity trends in EPIC-Potsdam (males). _____________  =  linear model using five observation points. __ __ __ __ __  =  leveling off model using five observation points.

**Figure 4:**
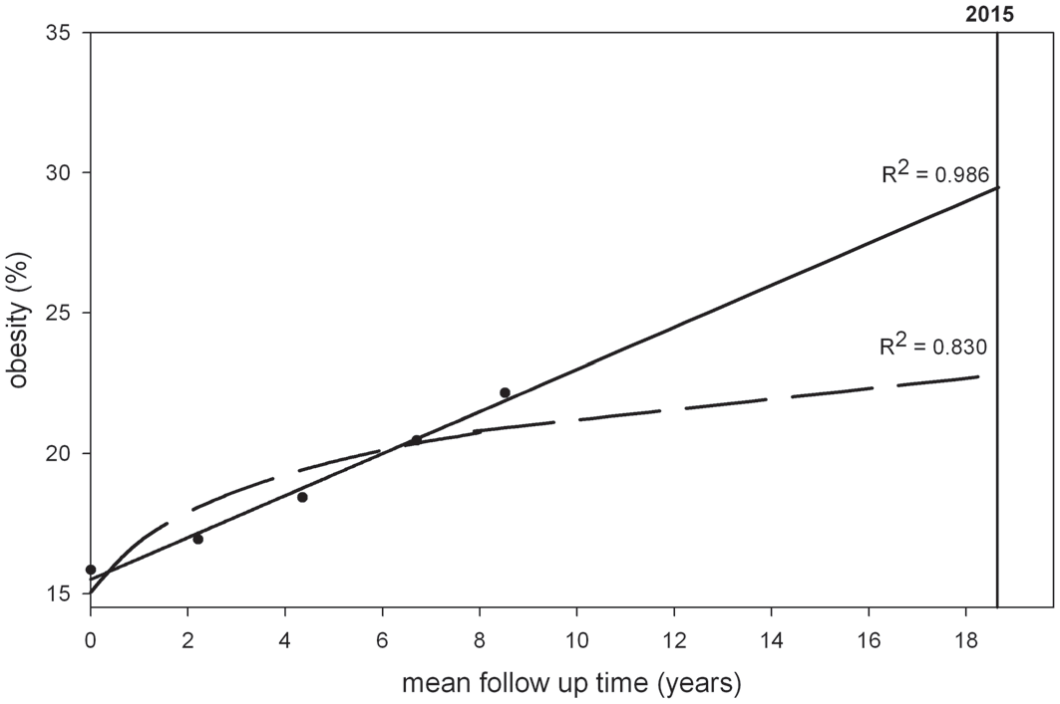
Obesity trends in EPIC-Potsdam (females). _____________  =  linear model using five observation points. __ __ __ __ __  =  leveling off model using five observation points.

Subjects of the EPIC-Potsdam cohort which served as a test population to check the linearity of the obesity trends had a mean age of 50.2 years (quartile range: 42.4–58.0 years) and a mean BMI of 26.1 kg/m^2^ (quartile range: 23.2-28.4 kg/m^2^) at baseline, and were followed for 8.6 years on average (quartile range: 7.8–9.3 years). The prevalence of obesity amounted to 17% and 16% at baseline and increased to 25% and 22% at the fourth follow-up for men and women, respectively (**Table 3**). It appeared that among men the increase in obesity prevalence during the last years of follow-up was less strong than in the initial period of observation. Thus, the log transformed model fitted the data slightly better to the EPIC Potsdam data than a linear model among men (**Figure 3**). In contrast, in women obesity prevalence increased in a relatively linear way over 8 years of follow-up (**Figure 4**). In general, the observation that obesity prevalences increases linearly in women and more non-linearly in men was independent of age at recruitment as this was also found in separate age groups (e.g. 40-49, 50-59, 60-65).

**Table 3:** Trends in obesity prevalence (BMI ≥ 30 kg/m^2^) in EPIC-Potsdam.

Measurement (year)	Baseline (1994–1998)	Follow Up 1 (1997–2001)	Follow Up 2 (1999–2003)	Follow Up 3 (2001–2005)	Follow Up 4 (2004–2008)
	Obesity [%]	Obesity [%]	Obesity [%]	Obesity [%]	Obesity [%]
**Men** *(n = 8 438)*					
EPIC-Potsdam	16.6	20.0	22.1	23.0	24.6
Application of linear model a	17.5 (16.7–18.3)^b^	19.3 (18.5–20.0)	21.1 (20.4–21.9)	23.3 (22.4–24.1)	25.1 (24.2–26.0)
Application of leveling off model a	16.4 (15.6–17.2)	20.2 (19.4–20.9)	22.0 (21.2–22.8)	23.4 (22.5–24.2)	24.2 (23.3–25.1)
**Women** *(n = 13 791)*					
EPIC-Potsdam	15.8	16.9	18.4	20.5	22.2
Application of linear model a	15.6 (15.0–16.2)	17.1 (16.5–17.7)	18.7 (18.1–19.3)	20.5 (19.9–21.1)	22.0 (21.3–22.7)
Application of leveling off model a	15.1 (14.5–15.7)	18.0 (17.4–18.5)	19.4 (18.8–20.0)	20.4 (19.8–21.0)	21.0 (20.4–21.7)

a Probability of being obese was modeled in dependency of follow-up time and age at recruitment using logistic regression.

b 95% confidence intervals are shown in parentheses and were calculated by bootstrapping.

(10 000 samples were used for men and women, respectively).

## Discussion

In the Diogenes cohort, the prevalence of obesity increased relatively independent of initial age in all study centers during follow-up. If the observed trend in increase of obesity during follow-up was linearly projected to the year 2015, an obesity prevalence of about 30% is expected in the participants aged 40–65 years at baseline in this cohort, which is similar to the situation in the US nowadays. The leveling off model predicted also considerable obesity prevalence in 2015, but with a much lower value compared to the linear model, with a mean estimate of about 20% among person aged 40–65 years initially of the Diogenes cohort.

The data and conclusions about observed and projected obesity prevalences have been derived from a longitudinal study. Conversely, publications dealing with the development of the obesity epidemic over time are mainly based on cross-sectional data [2], [10], [11], [12]. However, cross-sectional data reflect only the situation at a specific time across the age groups. For example, they often suggest that obesity rates peak between the ages of 50 to 60 years in most developed and developing countries. Thereafter, a drop of the rates is observed [2]. However, this drop in the prevalence does not necessarily mean that elderly subjects loose weight. Instead, it could be the result of birth cohort effects and reflect the fact that older birth cohorts had not gained similar weight with age as birth cohorts being born in a later time period. To overcome this problem of birth cohort effects making cross-sectional data based on one-point-in-time estimates less interpretable, longitudinal data are necessary: e.g. multiple sequential cross-sectional surveys with several birth cohorts and several specific ages or preferably prospective cohort studies. Despite this, longitudinal data on obesity trends are rarely found in the literature compared to cross-sectional data. Grinker et al. for example found that obesity was strongly related to age during a follow-up period of 15 years. The largest increase was observed among the 30-44 year old subjects, whereas subjects aged 60 years and older at baseline remained weight stable [19]. In our study population we also observed a relatively strong weight increase in young age groups. However, in contrast to Grinker et al., we could observe an increase in weight also in the age group of 65–75 years between the baseline and follow-up examination.

Moreover, literature about future prediction of obesity is rare and research groups that have investigated future trends in obesity based their projections on repeated survey data [7], [8], [9] [20]. These data is based on a random sample of different individuals at each time period; in contrast to our study that observed individuals of a wide age range over years. However, in contrast to repeated independent surveys, a prospective cohort is ageing during the observation time. To consider the impact of age on future trends in obesity prevalences, age at recruitment was included as a covariate into the regression models. Furthermore, we stratified by baseline age group to evaluate trends in obesity among different age groups.

In the following we compared our study results with attempts of other researchers to predict obesity prevalences in the future based on repeated cross-sectional surveys. Kelly et al. projected the global burden of obesity all over the world in 2030 [7]. Overall, 9.8% of the world's adult population was obese in 2005 (7.7% in men and 11.9% in women). Based on regional secular trends in obesity prevalence estimated from past data, population growth and demographic shifts, they predicted an increase of obesity prevalence of up to 19.7% in 2030. However, obesity prevalences are differing strongly between world regions. For the established market economies which comprise developed and industrialized high income countries the estimated prevalence of obesity in 2030 amounts to 36%. Wang et al. focused on the projection of future trends in the United States based on repeated nationwide survey data (National Health and Nutrition Examination Study, NHANES) collected between 1970 and 2004 [8]. They reported that 32.3% of the American adults were obese in 2004 and that this proportion will increase up to 51.1% in 2030 based on the assumption that trends will continue linearly. The projected prevalence amounts to 37.4% in 2010 and 44.2% in 2020. These prognoses reflect a worse situation than in Europe which is due to considerably higher initial obesity prevalence.

To address the limitations of linear predictions based on cross-sectional data, Wang et al. conducted a further analysis using fitted regression models stratified by sex and race based on the assumption that the cohort is aging 10 years to project shifts in BMI distribution of the 1999-2000 NHANES population to 2010 [20]. These projections were also validated with data from two longitudinal studies (Nurses' Health Study and Health Professional Follow-Up Study) making this approach comparable to our analyses. The expected prevalence of obesity for 2010 was 35% and 36% for white men and women, respectively. Zaninotto et al. investigated different scenarios to project the prevalence of adult obesity in England from 1993-2004 to 2012 based on repeated cross-sectional surveys [9]. Based on the linear trend, the prevalence of obesity among 35–74 year old individuals increased from 15.4–17.8% (men) or 17.9-22.8% (women) in 1993 to 35.1–36.4% for males and 30.6–35.9% for females in 2012. In contrast, the projection based on the best fitting curve allowing to consider a slowing down in the obesity trend lead to prevalences of 28.0–29.8% in men and 25.8–32.0% in women in this age group. As in our analysis, the non-linear modeling resulted in considerably lower estimates than the linear prediction which is not surprising. Similar to us Zaninotto et al. investigated alternatives to the linear model. We also decided to investigate a linear and non-linear scenario to project the situation in the near future, for the year 2015. We could show that differences in linear and non-linear projections could be huge if the observation period was short and the projection period long. Compared with Zaninotto et al., our projections based on the leveling off model appeared to be moderate. However, our prevalence data do not represent a specific region or country and are not representative for the total population.

Overall, the non-linear (leveling off) approach of prediction seems to be more realistic, whereas the linear scenario is more likely to result in an over-estimation of future obesity prevalences. It is likely that age-associated diseases will lead to a leveling off in future weight gain and thus, obesity trends might slow down. Moreover, as BMI reaches a high level, the risk for obesity-related diseases such as diabetes mellitus type 2 or metabolic syndrome increases considerably. Thus, severe obese subjects are forced to control their weight because of medical reasons. Therefore, one cannot assume that people gain weight in a linear way during their whole life. There is also some evidence in the literature based on repeated survey data that the trend in obesity prevalence, which was increasing nearly linearly in the past decades, is now showing a leveling off [21], [22], [23]. This fact in turn adds further support to the leveling off approach of prediction.

Besides investigating different scenarios for future projection a further strength of our study is the large sample size covering cohorts from five European countries and a total of 97,942 participants. Some differences in methodologies used to collect data on body weight might have affected the results. Therefore, we corrected body weight for clothing differences and self-reporting using the equations proposed by Haftenberger et al [18].

We took BMI as a measure of obesity, which can be discussed because it does not take body composition, i.e. fat and lean body mass, into account. However, several studies have shown that even without body weight changes, the amount of body fat significantly increases with age. That is why a BMI ≥ 30 kg/m^2^ is associated with an excess of body fat in any case, thus being an acceptable measure of obesity also in the elderly. Another measure of total body fat is waist circumference, which has been shown to be strongly related to visceral fat with a cut-off for adults of 102 cm in men and 88 cm in women to indicate abdominal obesity. Using these cut-offs led to a great proportion of elderly persons (60+) having high risk values of waist circumference [24]. However, these waist circumference cut-off points still need to be validated as predictors of morbidity and mortality in older ages [25].

One weakness of our study is that only two measurements of anthropometric values were available for each subject in the Diogenes cohort besides the cohort from Potsdam. Therefore, we also calculated trends until 2015 in the EPIC-Potsdam dataset where five measurements per participant were available to investigate which of the two prediction models (linear *vs.* non-linear) gives the better model fit. Furthermore, we compared models using five data points with models using two measurements to validate our projections. The predicted prevalence estimates for 2015 of these two different models showed a relatively good agreement (data not shown). Thus, we can draw the conclusion that also two observation points might be sufficient for a valid future projection.

However, the results of this study could also be biased by several factors such as selection bias or differential loss-to-follow-up bias. This implies that in general those who are obese or have a poor state of health are less likely to participate in a study for the whole period of time [26]. Hence, our data might underestimate real obesity prevalence trends even though already sizeable increases in obesity prevalence during life course across all study centers were observed. Furthermore, self-reporting of BMI can lead to under-estimations. As already mentioned, we applied correction equations to address this issue. Moreover, the same trends in obesity prevalence were observed in the cohorts that have measured data indicating that it might be valid to rely on corrected self-reported data. A further limitation of the study is that projections are based on a number of assumptions; some of them were simplified scenarios e.g. that the current trend will continue. Potential future policy-, environmental- and behavioral changes may disprove our predictions.

Since 2015 is in the near future and the study cohort is still on follow-up, it is possible to check whether our predictions had been valid in a few years. This will be an excellent opportunity to evaluate the accuracy of the linear and the leveling off model for the prediction of future obesity prevalences. Besides verifying our own predictions, further research is needed to investigate the development of weight with increasing age, especially in high age groups using a longitudinal study design.
